# Relationship between cardiovascular risk and lipid testing in one health care system: a retrospective cohort study

**DOI:** 10.1186/s12913-015-0884-2

**Published:** 2015-07-23

**Authors:** Robert J. Reid, Melissa L. Anderson, Paul A. Fishman, Jennifer B. McClure, Ron L. Johnson, Sheryl L. Catz, Beverly B. Green

**Affiliations:** Group Health Research Institute, 1730 Minor Avenue, Suite 1600, Seattle, WA 98122 USA; Group Health Physicians, Seattle, WA USA; Betty Irene Moore School of Nursing, University of California Davis, 4610 X Street, Sacramento, CA 95817 USA

**Keywords:** Cholesterol testing, Quality of care, Cardiovascular disease risk

## Abstract

**Background:**

The US Preventive Services Taskforce (USPSTF) recommends routine lipid screening beginning age 35 for men [1]. For women age 20 and older, as well as men age 20–34, screening is recommended if cardiovascular risk factors are present. Prior research has focused on underutilization but not overuse of lipid testing. The objective is to document over- and under-use of lipid testing in an insured population of persons at low, moderate and high cardiovascular disease (CVD) risk for persons not already on statins.

**Methods:**

The study is a retrospective cohort study that included all adults without prior CVD who were continuously enrolled in a large integrated healthcare system from 2005 to 2010.

Measures included lipid test frequency extracted from administrative data and Framingham cardiovascular risk equations applied using electronic medical record data. Five year lipid testing patterns were examined by age, sex and CVD risk. Generalized linear models were used to estimate the relative risk for over testing associated with patient characteristics.

**Results:**

Among males and females for whom testing is not recommended, 35.8 % and 61.5 % received at least one lipid test in the prior 5 years and 8.4 % and 24.4 % had two or more.

Over-testing was associated with age, race, comorbidity, primary care use and neighborhood income. Among individuals at moderate and high-risk (not already treated with statins) and for whom screening is recommended, between 21.4 % and 25.1 % of individuals received no screening in the prior 5 years.

**Conclusions:**

Based on USPSTF lipid screening recommendations, this study documents substantial over-testing among individuals with low CVD risk and under-testing among individuals with moderate to high-risk not already on statins. Opportunity exists to better focus lipid screening efforts appropriate to CVD risk.

## Background

Because hyperlipidemia is a leading risk factor for cardiovascular disease (CVD) that can be reduced by early intervention, the US Preventive Service Task Force (USPSTF) recommends routine lipid screening beginning at age 35 for men [1]. For women age 20 and older, as well as men age 20–34, screening is only recommended for persons at increased risk (*i.e.*, diabetes, hypertension, tobacco use, obesity, previous personal history of CVD, or premature family history of CVD) because, without these risks, the absolute 10-year CVD risk is small regardless of lipid levels. The USPSTF also recommends repeat screening for individuals with CVD risk factors and lipid lowering agents based on Framingham CVD risk projections.

A recent guideline from the American College of Cardiologists and American Heart Association (ACC-AHA) recommends universal lipid screening begin earlier at age 20 for both men and women [2]. They also recommend using an updated global CVD risk calculator to identify young adults at high long-term (30 year) risk of cardiac mortality. Estimates of 30-year risk might be useful for those under age 40 with established risk factors, but screening in this age group remains controversial because there is no evidence that long-term use of statins improves CVD outcomes. The ACC-AHA guideline also encourages lipid screening using the updated risk calculator and recommends statins for adults ages 40–75 with a 7.5 % 10-year CVD risk or higher.

While older guidelines endorsed regular monitoring of low density lipoprotein (LDL) for those on statins, the new guideline omits this recommendation.

Over the last several decades, there has been considerable attention on underutilization of cholesterol screening [3, 4] and the under-treatment of hyperlipidemia in persons at risk [5, 6].

Latest published data from the Behavioral Risk Factor Surveillance Survey show use of cholesterol screening is increasing in persons aged 18 and older, rising from 72.7 % to 76.0 % between 2005 and 2009 [7]. In addition to underuse, attention in the research and policy community has focused on overuse of preventive and other services where expenses and risks outweigh the benefits.

Research confirms that overuse is widespread [8, 9], not only resulting in unnecessary costs from testing and downstream expenditures, but also in psychological and other risks from over-diagnosis [10]. Because of the risks involved, most research on overuse of screening and monitoring has focused on colorectal, breast, cervical and prostate cancer screening [8, 9]. There has been little attention to overutilization of lipid screening, particularly in persons who are at low-risk. One study of Medicare enrollees [11], almost all of whom are at moderate- or high-risk, found that multiple testing of lipids was common and likely unnecessary (*i.e.*, 11.9 % of patients receiving three or more tests in a 1 year period). To our knowledge, no studies have examined overuse of lipid screening among low-risk or younger individuals, nor the characteristics that are associated with overuse. However, this examination is relevant. Identifying the under-use of recommended screenings could improve health outcomes for those at moderate- or high-risk, while identifying the over-use of screenings among persons at low-risk could point to opportunities for lowering population-level health care costs and reducing patient burden.

This investigation is part of the e-Care for Heart Wellness Study (RC1HL100590-01) [9] and focuses on the patterns of lipid testing among persons without prior CVD. The purpose of this study is to document over- and under-use of lipid testing as recommended by the USPSTF among an insured population including persons at low, moderate and high CVD risk. We also examine characteristics associated with over-testing among low risk persons.

## Methods

Cohort participants received care from Group Health, an integrated healthcare system in Washington State that provides coverage and care to approximately 405,000 persons in an integrated group practice. Participants were adults aged 25–79 who were continuously enrolled at Group Health for 5 years between April 1, 2005 and March 31, 2010. Continuous enrollment was defined as having no gap greater than 45 days. Patients were excluded if they also had less than one primary care visit in the 2 years between April 1, 2008 and March 31, 2010 since the cardiovascular risk estimation relies on data derived from healthcare encounters. Patients were also excluded if they had a previous CVD diagnosis (*i.e.*, myocardial infarction, angina, coronary surgery, cerebrovascular occlusion, transient ischemic attack, congestive heart failure and peripheral vascular disease), as lipid testing guidelines are different for those with existing CVD [2, 6]. We excluded persons with other significant diseases including cancer (except non-melanoma skin cancer), end-stage renal disease, hepatic failure and dementia because cholesterol testing may not be clinically relevant for this group. Finally, we also excluded persons who were dispensed 3-hydroxy-3-methylglutaryl-coenzyme A (HMG-CoA) reductase inhibitors (statins, because of the recommendation for lipid monitoring prior to 2013 [9]) and isotretinoin (one or more prescription fills in the study period).

To construct the study variables, we extracted data from Group Health’s electronic clinical and administrative data systems. These databases capture all care provided to enrollees at Group Health facilities and from external claims. Demographic variables (including age, sex, race, zip code and health plan) were extracted from health plan enrollment records as of April 1, 2010. Using methods developed by Krieger and colleagues [12], we created ecological-level income and education variables by aggregating zip codes into census tracks and merging them with median household income and education Census variables. To define rural and urban status, we used the rural–urban commuting area code schema (RUCAs, version 2.0) that reflect relative geographic densities based on population counts and community patterns [13]. To describe our population’s use of primary care, we counted the average number of primary care visits per year (to primary care physicians, nurse practitioners and physician assistants) across the 5 years. Finally, we counted the number of lipid tests on different days performed during the 5 year study period.

Biometric, laboratory and tobacco use data were extracted from the patient’s clinical data based on the most recent recorded value during the study period. In addition to continuous measures, body mass index was classified as normal weight (<25.0), overweight (25.0–29.9) and obese (≥30 kg/m^2^). Systolic blood pressure was classified as ≤ 140, 141–160, 161–180 and >180 mm/Hg. Lipid levels were based on the most recent value recorded and were aggregated into clinically-relevant categories of <200, 200–239 and ≥240 mg/dl for total cholesterol (TC); <40, 40–59 and ≥60 mg/dl for high density lipoprotein (HDL); and <100, 100–129, 130–159, 160–189 and ≥ 190 mg/dl for LDL. Tobacco status was classified as current smoker or non-smoker using the tobacco vital sign collected at the most recent visit. Diabetes was defined as having: two or more filled prescriptions for insulin or oral agents; one inpatient or two outpatient diagnoses of diabetes at any time during their enrollment at Group Health; a fasting glucose >126 mg/dl confirmed by a second out-of range test within 1 year; or a random glucose > 200 mg/dl also confirmed by a second test within 1 year. Treated hypertension was determined by looking for any filled prescription for an antihypertensive in the prior year. To measure comorbidity, we used Resource Utilization Bands (RUBs), the terminal categories of the Adjusted Clinical Group (ACG) system [14]. The ACG system is widely used to adjust for differences in case-mix among populations receiving health services in outpatient settings [15, 16].

Using our prior methods [17], we combined age, sex, systolic blood pressure, treatment for hypertension, diabetes status, smoking status, BMI, HDL and TC to calculate 10 year Framingham global CVD risk scores using both lab-based [6] and BMI-based approaches [17]. As recommended by the Adult Treatment Panel- III of the National Cholesterol Education Program (ATP-III) [9], patients were classified into three CVD risk categories: low (<10 %), moderate (10–20 %) and high (>20 %) to reflect standards for lipid management during the study period. The new ACC-AHA guideline (promulgated after the study period) encourages statin use for persons aged 40–75 with CVD risk ≥7.5 %, so we also created a secondary binary risk variable at this threshold to use in sensitivity analyses. Since lipid testing was not performed on approximately 40 % of the population during the study period, we used the BMI-based risk score as our primary measure of CVD risk [17]. Our prior research found BMI- and lab-based scores to be concordant in 78.2 % of patients. When discordant, the BMI-based risk was almost always in a higher category, with less than 2 % of adults with a BMI-based 10-year CVD risk of <10 % being reclassified to moderate (10–20 %) or high-risk (>20 %) based on laboratory testing when both risking methods were available [17]. Lab-based risk scores were used to characterize risk for individuals with missing BMI-based measures (n = 698, 1.2 %).

Demographic characteristics and CVD risk factors were summarized separately for men and women. To compare observed testing patterns to guideline recommendations, we first computed the proportion of patients who received zero, one, two, or three or more lipid tests during the prior 5 year period, stratified by age, sex and CVD risk. Because the USPSTF also recommends lipid testing among all adults aged 20 and older with diabetes, hypertension, tobacco use, or obesity, we further examined testing patterns for persons with low CVD risk scores with and without any of these individual risk factors. While the USPSTF also recommends early testing for persons with a premature CVD family history, population-based family history data were not available which prevented our ability to examine testing relative to this risk factor. Regression models were used to investigate whether patient characteristics were associated with receiving lipid testing more frequently than USPSTF recommendations, among those at low-risk excluding persons with diabetes, hypertension, tobacco use, or obesity. Over-testing was defined for those at low CVD risk as one or more test in the past 5 years for women of any age or men age <35, or two or more tests for men over 35. Generalized linear models with a log link and Poisson error distribution were used to estimate the relative risk of over-testing associated with patient characteristics. All analyses were conducted using Stata/MP version 12.0 for Windows statistical software (Stata Corp. LP, College Station, Texas). All study methods were approved by the Group Health Human Subjects Review Committee.

## Results

**Fig. 1:**
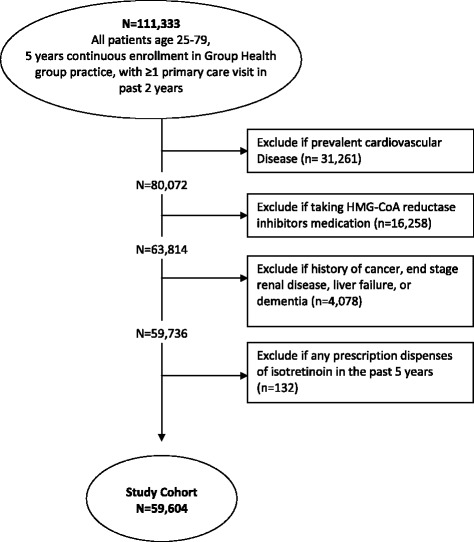
Study sample

Figure 1 outlines the selection of the study subjects. Of the 111,333 persons who were aged 25–79, continuously enrolled at Group Health, with at least one visit in the prior 2 years, we excluded 46.5 % because of prevalent CVD or other significant disease, or with filled prescriptions for HMG-CoA reductase inhibitors or isotretinoin. The final study cohort of 59,604 persons was 58.8 % female with a mean age of 51.1 years (SD 12.3) (Table 1.) As is characteristic of insured populations in Washington state, the vast majority were white (83.3 %), lived in urban settings (97.4 %), and had commercial health insurance (79.9 %.) Furthermore, a large portion lived in neighborhoods which were more than 70 % college educated (45.6 %) but with median household incomes less than $60,000 per year (68.7 %) using 2000 census data. No large differences in demographics were apparent between men and women.

**Table 1:** Population characteristics

	Men	Women
Total, N (row%)	24,540 (41.2)	35,064 (58.8)
	n (col%)	n (col%)
Age		
25–34	2,306 (9.4)	3,697 (10.5)
35–44	5,290 (21.6)	7,242 (20.7)
45–54	7,133 (29.1)	9,946 (28.4)
55–64	6,700 (27.3)	9,063 (25.9)
65–79	3,111 (12.7)	5,116 (14.6)
Cardiovascular risk^a^		
Low (<10 %)	7,661 (38.9)	24,192 (77.1)
Moderate (10–20 %)	6,290 (31.9)	5,378 (17.1)
High (>20 %)	5,745 (29.2)	1,798 (5.7)
Missing	4,844 [19.7]	3,696 [10.5]
Diabetes	552 (2.3)	710 (2.0)
BP-lowering medications, any fill in past year	4,870 (19.9)	6,477 (18.5)
Systolic blood pressure		
≤140	18,265 (84.7)	28,956 (88.6)
141–160	2,817 (13.1)	3,103 (9.5)
161–180	438 (2.0)	537 (1.6)
>180	56 (0.3)	91 (0.3)
Missing	2,964 [12.1]	2,377 [6.8]
Total cholesterol		
<200	6,745 (46.5)	8,781 (41.0)
200–239	5,508 (38.0)	8,352 (39.0)
≥240	2,256 (15.6)	4,273 (20.0)
Missing	10,031 [40.9]	13,658 [39.0]
HDL cholesterol		
<40	2,534 (17.5)	770 (3.6)
40–59	8,135 (56.1)	7,569 (35.4)
≥60	3,838 (26.5)	13,060 (61.0)
Missing	10,033 [40.9]	13,665 [39.0]
LDL cholesterol		
<100	2,207 (20.7)	3,721 (24.1)
100–129	3,945 (37.1)	5,808 (37.6)
130–159	3,094 (29.1)	4,049 (26.2)
160–189	1,101 (10.3)	1,467 (9.5)
≥190	302 (2.8)	401 (2.6)
Missing	13,891 [56.6]	19,618 [56.0]
BMI		
<25	4,761 (22.8)	12,481 (38.2)
25–29.9	9,220 (44.1)	9.646 (29.5)
≥30	6,913 (33.1)	10,545 (32.3)
Missing	3,646 [14.9]	2,392 [6.8]
Current smoker	2,188 (13.5)	3,130 (9.1)
Missing	843 [3.4]	587 [1.7]
RUB		
0 = No diagnoses	2,687 (11.0)	2,097 (6.0)
1 = Low	3,519 (14.3)	4,158 (11.9)
2	4,697 (19.1)	5,248 (15.0)
3	11,929 (48.6)	19,557 (55.8)
4	1,355 (5.5)	3,450 (9.8)
5 = High	353 (1.4)	554 (1.6)
Health plan		
Medicare	3,309 (13.5)	5,382 (15.4)
Medicaid/Basic Health	315 (1.3)	673 (1.9)
Commercial	19,945 (81.3)	27,699 (79.0)
Private pay	971 (4.0)	1,310 (3.7)
Annual average primary care visits, past 5 years		
<1	8,257 (33.7)	6,572 (18.7)
1 to <2	9,326 (38.0)	12,055 (34.4)
2 to <3	4,130 (16.8)	8,255 (23.5)
≥3	2,827 (11.5)	8,182 (23.3)
Residence		
Rural	660 (2.7)	914 (2.6)
Urban	23,880 (97.3)	34,150 (97.4)
Race		
White	14,866 (84.0)	25,805 (82.8)
Asian	1,418 (8.0)	3,105 (10.0)
Black	916 (5.2)	1,301 (4.2)
Mixed race/Other	490 (2.8)	951 (3.1)
Missing	6,850 [27.9]	3,902 [11.1]
Neighborhood-level variables		
Median household income		
<$40,000	5,371 (21.9)	7,643 (21.9)
$40,000 to <$60,000	11,394 (46.5)	16,410 (46.9)
$60,000 to <$80,000	5,606 (22.9)	8,014 (22.9)
≥$80,000	2,114 (8.6)	2,913 (8.3)
Missing	55 [0.2]	84 [0.2]
Education, % with some college		
<50 %	2,687 (11.0)	3,953 (11.3)
50 % to <60 %	4,096 (16.7)	5,968 (17.1)
60 % to <70 %	6,407 (26.2)	9,215 (26.3)
70 % to <80 %	5,973 (24.4)	8,554 (24.5)
≥80 %	5,319 (21.7)	7,289 (20.8)
Missing	58 [0.2]	85 [0.2]

^a^CVD Risk score based on BMI-based risk calculations. When BMI-risk was missing, we used lab-based risk calculations (n = 698). The CVD risk equations are as follows:

BMI-based CVD Risk Equations:

*Male, not treated with antihypertensive medications*

100*(1-(0.88431^(exp([3.11296*ln(AGE) + 0.79277*ln(BMI) + 1.85508*ln(SBP) + 0.70953*SMOKE + 0.53160*DIAB] -23.9388))))

*Male, treated with antihypertensive medications*

100*(1-(0.88431^(exp([3.11296* ln(AGE) + 0.79277* ln(BMI) + 1.92672* ln(SBP) + 0.70953* SMOKE + 0.53160* DIAB] -23.9388))))

*Female, not treated with antihypertensive medications*

100*(1-(0.94833^(exp([2.72107* ln(AGE) + 0.51125* ln(BMI) + 2.81291* ln(SBP) + 0.61868* SMOKE + 0.77763* DIAB] -26.0145))))

*Female, treated with antihypertensive medications*

100*(1-(0.94833^(exp([2.72107* ln(AGE) + 0.51125* ln(BMI) + 2.88267* ln(SBP) + 0.61868* SMOKE + 0.77763* DIAB] -26.0145))))

Lab-based CVD Risk Equations:

*Male, not treated with antihypertensive medications*

100*(1-(0.88936^(exp([3.06117*ln(AGE) + 1.12370*ln(TC)+−0.93263*ln(HDL) + 0.65451*SMOKE + 0.57367*DIAB + 1.93303* ln(SBP)]-23.9802))))

*Male, treated with antihypertensive medications*

100*(1-(0.88936^(exp([3.06117*ln(AGE) + 1.12370*ln(TC)+−0.93263*ln(HDL) + 0.65451*SMOKE + 0.57367*DIAB + 1.99881* ln(SBP)]-23.9802))))

*Female, not treated with antihypertensive medications*

100*(1-(0.95012^(exp([2.32888*ln(AGE) + 1.20904*ln(TC)+−0.70833*ln(HDL) + 0.52873*SMOKE + 0.69154*DIAB + 2.76157* ln(SBP)] -26.1931))))

*Female, treated with antihypertensive medications*

100*(1-(0.95012^(exp([2.32888*ln(AGE) + 1.20904*ln(TC)+−0.70833*ln(HDL) + 0.52873*SMOKE + 0.69154*DIAB + 2.82263* ln(SBP)] -26.1931))))

Based on BMI-based Framingham calculations, most women (77.1 %) were classified as low-risk (<10 %), 17.1 % were at moderate-risk (10–20 %), and only 5.7 % were at high-risk (>20 %) and not already prescribed statin medications. However, among men, the proportions at moderate (31.9 %) and high-risk (29.2 %) not already prescribed statins were substantially higher. While the proportions of men and women whose last recorded systolic blood pressures was >140 mmHg were similar (15.3 % *vs.* 11.4 %), men were more likely to be smokers (13.5 % *vs.* 9.1 %) and be overweight or obese (77.2 % *vs.* 61.8 %.) No large differences were apparent in the proportions of men or women with diabetes (2.3 % *vs.* 2.0 %), or who had hypertension (19.9 % *vs.* 18.5 %.) When overall illness burden was summarized with RUB categories, the majority of the population had a moderate burden (RUB 3) (52.9 %) and only 9.6 % were classified with high or very high burdens (RUBs 4–5.) Expected differences between men and women in TC and HDL levels were apparent [18].

Given the difference in USPSTF lipid screening recommendations for men and women of different ages and risk levels [1], Table 2 presents the proportion of subjects who received lipid testing in the prior 5 years, by age, sex and CVD risk category. The table presents percentages of patients who received zero, one, two or three or more lipid tests during the prior 5 year period. For low-risk men less than age 35 years for whom cholesterol screening is not recommended [1], 37.6 % had at least one test in the prior 5 years and 10.7 % had two or more.

**Table 2:** Lipid testing in the past 5 years by sex, age & cardiovascular disease (CVD) risk

Row(%)	Low CVD risk (<10 %)					Moderate CVD risk (10-20 %)					High CVD risk (>20 %)					Cannot determine CVD risk
	Lipid tests					Lipid tests					Lipid tests					
a) Men																
Age	0	1	2	3+	Total	0	1	2	3+	Total	0	1	2	3+	Total	Total
25-34	62.4	26.9	7.4	3.3	1654	50.0	25.0	12.5	12.5	8	0.0	100.0	0.0	0.0	1	643
35-44	43.1	38.1	11.6	7.1	3478	36.9	34.9	16.2	12.1	464	33.3	19.4	16.7	30.6	36	1312
45-54	29.7	42.6	17.5	10.2	2332	27.8	37.1	20.8	14.3	2631	28.3	32.5	19.1	20.2	754	1416
55-64	25.0	40.3	23.5	11.2	196	21.8	37.2	23.3	17.6	2909	21.4	33.7	23.0	21.9	2478	1117
65-79	0.0	0.0	100.0	0.0	1	14.0	29.1	28.4	28.4	278	19.1	28.1	24.4	28.4	2476	356
Total	42.7	37.1	12.8	7.4	7661	25.1	36.7	21.9	16.3	6290	21.4	31.1	23.1	24.5	5745	4844
b) Women																
Age	0	1	2	3+	Total	0	1	2	3+	Total	0	1	2	3+	Total	Total
25-34	70.1	21.9	4.9	3.3	3259					0					0	438
35-44	47.0	35.2	11.6	6.3	6436	31.2	16.9	16.9	35.1	77	0.0	80.0	0.0	20.0	5	724
45-54	29.2	41.3	19.3	10.3	7993	29.0	34.4	20.1	16.5	780	18.1	22.3	22.3	37.2	94	1079
55-64	22.4	38.7	23.0	15.8	5485	21.2	33.9	26.3	18.6	2180	21.8	26.6	22.6	29.1	399	999
65-79	20.1	35.5	25.6	18.7	1019	21.7	32.4	24.6	21.3	2341	23.1	32.0	19.8	25.2	1300	456
Total	37.5	36.2	16.4	9.9	24192	22.7	33.1	24.5	19.7	5378	22.5	20.4	20.5	26.6	1798	3696

Yellow shading indicates more testing than recommended by the United States Preventive Services Task Force (USPSTF) for persons with low CVD risk. Green shading indicates less testing than recommended for persons at moderate- and high-risk and who are not already on statins. Cross-hatch lines signify risk categories with no study subjects

Among low-risk women of any age where screening is also not recommended, these percentages were greater, with 62.5 % having at least one test and 26.3 % having two or more in the prior 5 years. For persons at moderate-risk (10–20 %) who were not already on statins where the USPSTF recommends cholesterol testing at least once every 5 years [1], 25.1 % of men and 22.7 % of women had no cholesterol testing. Among persons at high-risk not already on statins where yearly testing is recommended, 21.4 % of men and 22.5 % of women had no record of lipid testing in the 5 years period. When we applied the new ACC-AHA guidelines [19], only a small minority of persons less than 40 shifted from low to higher risk (185/9,622, 1.9 %) when the new low-risk cut-point was applied (7.5 % *vs.* 10 % risk.) Using this new criterion, 40.6 % persons aged 40 and older at low-risk (<7.5 %) had one test in the prior 5 years and 28.8 % had two or more.

Because the USPSTF also recommends screening, regardless of aggregate CVD risk scores, for all adults 20 years or older who are obese, use tobacco, or have hypertension or diabetes, Table 3 presents the study’s main findings and displays testing for low-risk individuals with none (n = 20,162, 63.3 %) versus one or more or of these factors risks (n = 11,691, 36.7 %.) For low-risk men less than age 35 with no risks, 35.8 % had at least one test and 8.4 % and two or more.

**Table 3:** Lipid testing in the past 5 years for adults with low cardiovascular disease (CVD) risk (<10 %) by number of CVD risk factors

Row(%)	No risk factors					1-4 risk factors				
	Lipid tests					Lipid tests				
a) Men										
Age	0	1	2	3+	Total	0	1	2	3+	Total
25-34	64.2	27.4	6.4	2.0	891	60.3	26.3	8.5	4.9	763
35-44	41.6	40.2	11.6	6.6	2040	45.3	35.2	11.7	7.9	1438
45-54	29.3	43.3	18.0	9.4	1853	31.3	39.9	15.5	13.4	479
55-64	25.5	40.1	23.4	10.9	192	0.0	50.0	25.0	25.0	4
65-79	0.0	0.0	100.0	0.0	1					0
Total	40.4	39.0	13.5	7.0	4977	47.0	33.5	11.5	8.0	2684
b) Women										
Age	0	1	2	3+	Total	0	1	2	3+	Total
25-34	74.8	19.6	3.4	2.2	2017	62.3	25.5	7.2	5.0	1242
35-44	50.6	35.0	10.1	4.3	3730	42.1	35.4	13.6	8.9	2706
45-54	29.9	43.4	18.4	8.3	4708	28.1	38.1	20.7	13.1	3285
55-64	22.4	40.4	22.1	15.2	3869	22.5	34.8	25.3	17.5	1616
65-79	20.4	37.2	24.4	18.0	861	18.4	26.6	32.3	22.8	158
Total	38.5	37.1	15.6	8.8	15,185	35.9	34.8	17.7	11.7	9007

Risk factors include diabetes, treated hypertension, current smoker, body mass index >30. Yellow shading indicates more testing than recommended by the United States Preventive Services Task Force (USPSTF) for persons with no risk factors. Green shading indicates less testing than recommended for persons with one or more risk factors and who are not already on statins. Cross-hatch lines signify risk categories with no study subjects

Similarly, for low-risk women of any age with no risks, 61.5 % had at least one test and 24.4 % had two or more.

Table 4 examines potential determinants of receipt of testing more frequently than recommended among low-risk persons (<10 % CVD risk) and with no individual risk factors. The table presents both unadjusted and adjusted relative risks. (The adjusted relative risks are adjusted for all of the other characteristics listed in the table.) For women, age was strongly associated with over-testing with low-risk women in all age categories being more likely than women aged 25–34 to be tested more frequently than guideline recommended (RR 2.04–3.16.) or men, age categories 35–44 and 45–54 were associated with lower likelihood of testing (RR 0.54–0.80) than for men aged 25–34. For both men and women, non-white race and averaging at least one primary care visits per year over the past five years were factors generally associated with a higher likelihood of testing in the multivariate models. While neighborhood education levels were not associated with over-testing among men or women, women in neighborhoods with higher median income had increased risk of over-testing. Significant comorbidity (RUB 3–5) was associated with over-testing among men but not women.

**Table 4:** Factors associated with lipid testing among adults with low cardiovascular risk (<10 %) and no CVD risk factors

	Men				Women			
	Total sample	Tested more than recommended	Unadjusted	Adjusted	Total sample	Tested more than recommended	Unadjusted	Adjusted
	N	n (%)	RR (95 % CI)	RR (95 % CI)	N	n (%)	RR (95 % CI)	RR (95 % CI)
Overall	4977	1266 (25.4)			15185	9341 (61.5)		
Age								
25–34	891	319 (35.8)	Referent	Referent	2017	508 (25.2)	Referent	Referent
35–44	2040	372 (18.2)	0.51 (0.45, 0.58)	0.54 (0.46, 0.63)	3730	1843 (49.4)	1.96 (1.81, 2.13)	2.04 (1.86, 2.24)
45–54	1853	508 (27.4)	0.77 (0.68, 0.86)	0.80 (0.70, 0.92)	4708	3301 (70.1)	2.78 (2.58, 3.01)	2.84 (2.60, 3.11)
55–64	192	66 (34.4)	0.97 (0.78, 1.20)	0.87 (0.68, 1.12)	3869	3004 (77.6)	3.08 (2.85, 3.33)	3.16 (2.89, 3.45)
65–79	1*	1* (100.0)	--	--	861	685 (79.6)	3.16 (2.91, 3.43)	3.01 (2.64, 3.43)
Race								
White	2779	679 (24.4)	Referent	Referent	11012	6926 (62.9)	Referent	Referent
Asian	469	181 (38.6)	1.58 (1.38, 1.80)	1.63 (1.43, 1.86)	1915	1356 (70.8)	1.13 (1.09, 1.16)	1.20 (1.16, 1.24)
Black	164	50 (30.5)	1.25 (0.98, 1.59)	1.30 (1.03, 1.65)	383	231 (60.3)	0.96 (0.88, 1.04)	1.09 (1.01, 1.18)
Other	81	17 (21.0)	0.86 (0.56, 1.32)	0.86 (0.55, 1.32)	301	171 (56.8)	0.90 (0.82, 1.00)	1.01 (0.92, 1.11)
RUB								
0 = No diagnoses	512	89 (17.4)	0.80 (0.64, 1.02)	0.93 (0.70, 1.23)	786	419 (53.3)	0.89 (0.82, 0.96)	0.89 (0.83, 0.96)
1 = Low	722	156 (21.6)	Referent	Referent	1879	1127 (60.0)	Referent	Referent
2	1212	301 (24.8)	1.15 (0.97, 1.36)	1.09 (0.89, 1.33)	2756	1622 (59.9)	0.98 (0.93, 1.03)	0.97 (0.93, 1.02)
3	2312	651 (28.2)	1.30 (1.12, 1.52)	1.21 (1.00, 1.45)	8382	5375 (64.1)	1.07 (1.03, 1.11)	1.01 (0.97, 1.05)
4 or 5 = High	219	69 (31.5)	1.46 (1.15, 1.85)	1.38 (1.05, 1.82)	1382	798 (57.7)	0.96 (0.91, 1.02)	0.98 (0.93, 1.04)
Primary care visits (annual avg)								
<1	1614	298 (18.5)	Referent	Referent	2546	1481 (58.2)	Referent	Referent
1 to <2	2159	568 (26.3)	1.42 (1.26, 1.61)	1.32 (1.14, 1.53)	5950	3721 (62.5)	1.08 (1.03, 1.12)	1.06 (1.02, 1.10)
2 to <3	789	254 (32.2)	1.74 (1.51, 2.01)	1.55 (1.30, 1.84)	3738	2387 (63.9)	1.10 (1.05, 1.14)	1.10 (1.05, 1.14)
≥3	415	146 (35.2)	1.91 (1.61, 2.25)	1.59 (1.30, 1.95)	2951	1752 (59.4)	1.02 (0.98, 1.07)	1.06 (1.02, 1.11)
Residence								
Rural	79	19 (24.1)	0.94 (0.64, 1.40)	1.11 (0.74, 1.65)	325	198 (60.9)	0.99 (0.91, 1.08)	1.04 (0.96, 1.13)
Urban	4898	1247 (25.5)	Referent	Referent	14860	9143 (61.5)	Referent	Referent
Health plan								
Medicare	60	18 (30.0)	1.17 (0.79, 1.73)	1.11 (0.73, 1.69)	966	755 (78.2)	1.29 (1.25, 1.34)	1.06 (0.96, 1.17)
Medicaid/Basic Hlth	83	19 (22.9)	0.89 (0.60, 1.33)	0.97 (0.66, 1.44)	326	168 (51.5)	0.85 (0.77, 0.95)	0.92 (0.84, 1.01)
Commercial	4610	1180 (25.6)	Referent	Referent	13194	7969 (60.4)	Referent	Referent
Private pay	224	49 (21.9)	0.85 (0.66, 1.10)	0.76 (0.54, 1.07)	699	449 (64.2)	1.06 (1.00, 1.13)	1.02 (0.97, 1.08)
Neighborhood income, median								
<$40,000	1138	287 (25.2)	Referent	Referent	2974	1439 (48.4)	Referent	Referent
$40,000–<$60,000	2349	588 (25.0)	0.99 (0.88, 1.12)	1.00 (0.86, 1.16)	6953	4340 (62.4)	1.29 (1.24, 1.34)	1.20 (1.15, 1.25)
$60,000–<$80,000	1088	263 (24.2)	0.96 (0.83, 1.11)	1.03 (0.86, 1.23)	3705	2444 (66.0)	1.36 (1.30, 1.42)	1.22 (1.17, 1.28)
≥$80,000	390	124 (31.8)	1.26 (1.06, 1.50)	1.24 (0.98, 1.56)	1512	1091 (72.2)	1.49 (1.42, 1.57)	1.29 (1.23, 1.37)
Neighborhood education, % with some college								
<50 %	471	135 (28.7)	Referent	Referent	1376	786 (57.1)	Referent	Referent
50 % to <60 %	722	180 (24.9)	0.87 (0.72, 1.05)	0.92 (0.74, 1.15)	2251	1285 (57.1)	1.00 (0.94, 1.06)	0.95 (0.90, 1.01)
60 % to <70 %	1290	321 (24.9)	0.87 (0.73, 1.03)	0.96 (0.78, 1.17)	3772	2251 (59.7)	1.04 (0.99, 1.10)	0.95 (0.90, 1.00)
70 % to <80 %	1276	318 (24.9)	0.87 (0.73, 1.03)	0.90 (0.73, 1.11)	3938	2497 (63.4)	1.11 (1.05, 1.17)	0.96 (0.91, 1.01)
≥80 %	1205	308 (25.6)	0.89 (0.75, 1.06)	0.90 (0.72, 1.13)	3806	2494 (65.5)	1.15 (1.09, 1.21)	0.93 (0.88, 0.98)

CVD risk factors include obesity (BMI > 30 kg/m2), tobacco use, diabetes and treated hypertension. *RR* relative risk, *RUB* Resource Utilization Band. Participant “*” was included with the 55–64 age group for regression model

## Discussion

Among persons at low-risk for CVD determined through the BMI-based Framingham calculations, no individual risk factors (obesity, tobacco use, hypertension, or diabetes), and under age 35 for men, a substantial proportion of men (35.8 %) and women (61.5 %) received cholesterol testing that is not recommended by the USPSTF. Similarly, among moderate- and high-risk individuals, 24.0 % and 21.6 % of these persons respectively had no lipid testing, reflecting under-screening as per guideline recommendations. Taken together, these findings suggest sizable gaps in targeting lipid screening across the risk spectrum that result in both missing prevention opportunities at one end and unnecessary screening at the other (particularly for women). While lipid testing is relatively inexpensive, there may be downstream consequences, particularly for those at low-risk, such as potential distress over an abnormal value [20], or the time clinicians use to explain results. Additionally provision of CVD risk information alone, does little to change risk [21]. Population identification of individuals likely to be at high-risk for CVD without lipid testing or not on statins would lead to more efficient and effective use of clinical resources. We were not able to determine CVD risk (with lab or BMI-based approach) on approximately 14 % of the study population suggesting that improvements in electronic vital sign data entry are needed to fully characterize risk and the need for cholesterol testing.

Many studies have explored factors related to underuse of lipid testing and CVD risk assessment. However less is known about the prevalence and factors associated with over use of lipid testing. Goodwin *et al.*, in a Medicare population, found that exposure to health referral region and care by multiple different physicians was associated with overtesting independent of indication for testing, co-morbidities and total physician visits [11]. Virini *et al.* found that almost one third of Veteran’s Administration patients with coronary heart disease and who had attained LDL goals had more than one LDL test over an 11-month time period [22].

Our study included low risk and younger patients. Among low-risk persons, factors that are generally associated with over-testing include advancing age and more primary care visits. This finding suggests that care providers may be more inclined to order, or patients request, lipid testing if they are older and make frequent visits, independent of their low risk status. In other words, providers and patients may be inclined to overestimate CVD risk and need for lipid screening, particularly for women, based on age and visit frequency, leading to greater attention to early and more lipid screening. This finding suggests that many providers are not consistently using risk markers and scoring algorithms to guide lipid screening as recommended by the USPSTF. This is not unexpected given that barriers exist to using risk calculators (*e.g.*, physicians often have to manually retrieve and enter the risk details) [23–26] and they can be difficult for patients to understand [21, 27]. Furthermore, while the national Choosing Wisely campaign has gathered momentum on reducing overuse of many services [28], cholesterol overtesting is not currently a core measure.

Our study has some notable limitations. First, it is important to note however, because we excluded persons taking statins, we likely underestimated the proportion of persons at moderate or high-risk who were appropriately screened. Rather, our under-use estimates should be interpreted as the proportion of moderate or high risk persons not currently taking a statin who remain unscreened. Second, our study relied on BMI- rather than traditional lipid-based CVD risk estimates to classify individuals into risk categories. While this can create misclassification bias, we believe that bias to be conservative with respect to over testing in low-risk people since when the scores differ, the BMI-based scores are almost always higher [17]. Third, since family history [29] and socioeconomic risk [30] are independent CVD predictors but are not incorporated into traditional calculators, physicians may be appropriately screening low-risk people with positive family histories or who come from impoverished areas. However, we believe this limitation to be minor since research finds adding these factors only make slight improvements to population-based risk categorization [30, 31]. Finally, since the study takes place in an integrated delivery system with a history of applying evidence-based prevention guidelines [32], our results likely underestimate the degree of mismatch between CVD risk and lipid testing to be found in other practice settings. The study participants were also largely white and had health insurance which may limit out study’s generalizability.

## Conclusion

Our study findings clearly highlight the opportunity to improve adherence to USPSTF screening guidelines.

In so doing, physicians may reduce unnecessary medical costs and burden for a significant subset of patients, while improving care for others.

## Abbreviations

ACC-AHA: American College of Cardiology-American Heart Association
ACG: Adjusted Clinical Group
ATP – III: Adult Treatment Panel – III
BMI: Body Mass Index
CVD: Cardiovascular Disease
HDL: High Density Lipoprotein
HMG-CoA: 3-hydroxy-3-methylglutaryl-coenzyme A
LDL: Low Density Lipoprotein
RUB: Resource Utilization Band
RUCA F: Rural–urban Commuting Area
TC: Total Cholesterol
USPSTF: US Preventive Services Task Force

## References

[1] United States Preventive Services Task Force. Screening for Lipid Disorders in Adults. 2008. http://www.uspreventiveservicestaskforce.org/uspstf/uspschol.htm. Accessed March 29, 2014.

[2] Stone NJ, Robinson J, Lichtenstein AH, *et al.* 2013 ACC/AHA guideline on the treatment of blood cholesterol to reduce atherosclerotic cardiovascular risk in adults: a report of the American College of Cardiology/American Heart Association Task Force on practice guidelines. J Am Coll Cardiol 2014: 63(25 - Part B):2889-2934. 10.1016/j.jacc.2013.11.002 24239923

[3] Trivedi AN, Grebla RC (2011). Quality and equity of care in the Veterans Affairs Health-Care System and in medicare advantage health plans. Medical Care.

[4] Yoon PW, Tong X, Schmidt SM, Matson-Koffman D. Clinical preventive services for patients at risk for cardiovascular disease,National Ambulatory Medical Care Survey, 2005-2006. Prev Chronic Dis 2011;8(2):A43. PMC3073436 21324257

[5] Waters DD, Brotons C, Chiang CW (2009). Lipid treatment assessment project 2: a multinational survey to evaluate the proportion of patients achieving low-density lipoprotein cholesterol goals. Circulation.

[6] National Heart Lung and Blood Institute, Boston University. Framingham Heart Study. 2011; http://www.framinghamheartstudy.org/risk-functions/coronary-heart-disease/hard-10-year-risk.php. Accessed June 23, 2011.

[7] Centers for Disease C, Prevention (2012). Prevalence of cholesterol screening and high blood cholesterol among adults–United States, 2005, 2007 and 2009. MMWR Morb Mortal Wkly Rep.

[8] Korenstein D, Falk R, Howell EA, Bishop T, Keyhani S (2012). Overuse of health care services in the United States: an understudied problem. Arch Intern Med.

[9] Expert Panel on Detection E, Treatment of High Blood Cholesterol in A (2001). Executive summary of the third report of the National Cholesterol Education Program (NCEP) expert panel on detection, evaluation and treatment of high blood cholesterol in adults (Adult Treatment Panel III). JAMA.

[10] Berwick DM, Hackbarth AD (2012). Eliminating waste in US health care. JAMA.

[11] Goodwin JS, Asrabadi A, Howrey B, Giordano S, Kuo YF (2011). Multiple measurement of serum lipids in the elderly. Med Care.

[12] Krieger N (1992). Overcoming the absence of socioeconomic data in medical records: validation and application of a census-based methodology. Am J Public Health.

[13] Hart LG, Larson EH, Lishner DM (2005). Rural definitions for health policy and research. American Journal of Public Health.

[14] The Johns Hopkins University (2011). The Johns Hopkins ACG System Technical Reference Guide, version 10.0.

[15] Weiner JP, Starfield BH, Steinwachs DM, Mumford LM (1991). Development and application of a population-oriented measure of ambulatory care case-mix. Med Care.

[16] Starfield B, Weiner J, Mumford L, Steinwachs D (1991). Ambulatory care groups: a categorization of diagnoses for research and management. Health Serv Res.

[17] Green BB, Anderson ML, Cook AJ (2012). Using body mass index data in the electronic health record to calculate cardiovascular risk. Am J Prev Med.

[18] Arsenault BJ, Rana JS, Stroes ES (2009). Beyond low-density lipoprotein cholesterol: respective contributions of non-high-density lipoprotein cholesterol levels, triglycerides and the total cholesterol/high-density lipoprotein cholesterol ratio to coronary heart disease risk in apparently healthy men and women. J Am Coll Cardiol.

[19] Goff DC, Lloyd-Jones DM, Bennett G, *et al.* 2013 ACC/AHA Guideline on the Assessment of Cardiovascular Risk: A Report of the American College of Cardiology/American Heart Association Task Force on Practice Guidelines. Circulation. 2014;129:S49-S73. 10.1161/01.cir.0000437741.48606.98 24222018

[20] Bonner C, Jansen J, Newell BR (2014). I don’t believe it, but i’d better do something about it: patient experiences of online heart age risk calculators. J Med Internet Res.

[21] Sheridan SL, Viera AJ, Krantz MJ (2010). The effect of giving global coronary risk information to adults: a systematic review. Arch Intern Med.

[22] Virani SS, Woodard LD, Wang D (2013). Correlates of repeat lipid testing in patients with coronary heart disease. JAMA Intern Med.

[23] Sposito AC, Ramires JA, Jukema JW (2009). Physicians’ attitudes and adherence to use of risk scores for primary prevention of cardiovascular disease: cross-sectional survey in three world regions. Curr Med Res Opin.

[24] Hobbs FD, Jukema JW, Da Silva PM, McCormack T, Catapano AL (2010). Barriers to cardiovascular disease risk scoring and primary prevention in Europe. QJM.

[25] van Steenkiste B, van der Weijden T, Stoffers HE, Grol R (2004). Barriers to implementing cardiovascular risk tables in routine general practice. Scand J Prim Health Care.

[26] McKillop A, Crisp J, Walsh K (2012). Barriers and enablers to implementation of a New Zealand-wide guideline for assessment and management of cardiovascular risk in primary health care: a template analysis. Worldviews Evid Based Nurs.

[27] van Steenkiste B, van der Weijden T, Timmermans D, Vaes J, Stoffers J, Grol R (2004). Patients’ ideas, fears and expectations of their coronary risk: barriers for primary prevention. Patient Educ Couns.

[28] Cassel CK, Guest JA (2012). Choosing wisely: helping physicians and patients make smart decisions about their care. JAMA.

[29] Myers RH, Kiely DK, Cupples LA, Kannel WB (1990). Parental history is an independent risk factor for coronary artery disease: the Framingham study. American Heart Journal.

[30] Woodward M, Brindle P, Tunstall-Pedoe H (2007). Adding social deprivation and family history to cardiovascular risk assessment: the ASSIGN score from the Scottish Heart Health Extended Cohort (SHHEC). Heart.

[31] Sivapalaratnam S, Boekholdt SM, Trip MD (2010). Family history of premature coronary heart disease and risk prediction in the EPIC-Norfolk prospective population study. Heart.

[32] Thompson R (1996). What have HMOs learned about clinical prevention services? An examination of the experience at Group Health Cooperative of Puget Sound. Milbank Q.

